# A proposal for virtual, telephone-based preoperative cognitive assessment in older adults undergoing elective surgery

**DOI:** 10.1186/s13741-022-00274-z

**Published:** 2022-08-18

**Authors:** Lisa Cooper, Sindhu Krishnan, Houman Javedan, Angela M. Bader, Samir Tulabaev

**Affiliations:** 1 Division of Aging, Brigham and Women’s Hospital, 75 Francis Street, Boston, MA USA; 2 Department of Anesthesiology, Perioperative Medicine and Pain, Brigham and Women’s Hospital, 75 Francis Street, Boston, MA USA; 3 Center for Surgery and Public Health, Brigham and Women’s Hospital, Boston, MA USA

**Keywords:** Preoperative medicine, Cognitive assessment, Virtual visits, Older adults, Elective surgery

## Abstract

**Objective:**

To assess the feasibility of administering the MoCA 5-minute test/Telephone (T-MoCA), an abbreviated version of the Montreal Cognitive Assessment to older adults perioperatively

**Design:**

A feasibility study including patients aged ≥ 70 years scheduled for surgery from December 2020 to March 2021

**Setting:**

Preoperative virtual clinic

**Patients:**

Patients ≥70 years undergoing major elective surgery

**Intervention:**

A study investigator called eligible patients prior to surgery, obtained consent, and completed the preoperative cognitive assessment. Follow-up assessment was completed 1-month postoperatively, and participating clinicians were surveyed at the completion of the study.

**Measurements:**

An attention test, T-MoCA, Activities of Daily Living (ADL), Instrumental Activities of Daily Living (IADL), and Generalized Anxiety Disorder 2-item (GAD-2)

**Main results:**

Overall, 37/40 (92.5%) patients completed the pre- and post-operative assessments. The cohort was 50% female, white (97.5%), with a median age of 76 years (interquartile range (IQR) 73-79), and education level was higher than high school in 82.5% of patients. Preoperatively, the median number of medications was 8 (IQR 7-11), 27/40 (67.5%) had medications with anticholinergic effects, and 6/40 (15%) had benzodiazepines. Median completion time of the phone assessment was 10 min (IQR 8.25-12) and 4 min (IQR 3-5) for the T-MoCA with a median T-MoCA score of 13 (IQR 12-14). Most patients (37/40) completed the post-operative assessment, and 6/37 (16.2%) reported they had experienced a change in memory or attention post-operatively. Clinician’s survey reported ease and feasibility in performing T-MoCA as a preoperative cognitive evaluation.

**Conclusion:**

Preoperative cognitive assessment of older adults using T-MoCA over the phone is easy to perform by clinicians and had a high completion rate by patients. This test is feasible for virtual assessments. Further research is needed to better define validity and correlation with postoperative outcomes.

## Introduction

Undiagnosed preoperative cognitive impairment is associated with increased postoperative complications including postoperative delirium, postoperative cognitive dysfunction, non-home discharge, increased length of stay, and increased risk of mortality (Decker et al. 2020; Culley et al. 2017). These complications can negatively impact the benefits associated with the surgery (Arias et al. 2020) and add significant cost as well. However, preoperative cognitive assessments are performed in less than 10% of the older adults presenting to surgery (Mahanna-Gabrielli et al. 2019). The potentially detrimental consequences of postoperative cognitive impairment in this population have led to calls for action by multiple professional groups, including the American Society of Anesthesiologists (ASA) with their Perioperative Brain Health Initiative, as well as the American Geriatric Society and American College of Surgeons (Decker et al. 2020; Mahanna-Gabrielli et al. 2019; Berian et al. 2017).

There are multiple cognitive assessments available, including the Mini-Cog, Mini-Mental State Exam (MMSE), and Montreal Cognitive Assessment (MoCA) (Arias et al. 2020). Prior to the COVID-19 pandemic, we routinely performed preoperative cognitive testing with the Mini-Cog during the in-person visits to our preoperative assessment clinic (Sherman et al. 2019). However, with the start of the COVID-19 pandemic in early 2020, our pre-op clinics performed virtual visits instead of in-person assessments, making the Mini-Cog no longer possible. Knowing the importance of preoperative cognitive assessment led us to trial the MoCA 5-minute test/Telephone (which we will refer to as the T-MoCA) in older adults who are candidates for major surgery. We specifically chose this tool because it can be performed remotely during a short time period (5 min) by trained multidisciplinary clinicians and because of its reliability in detecting mild cognitive impairment (Wong et al. 2015).

Our goal is to provide a way to incorporate perioperative cognitive evaluations during these challenging times of the COVID-19 pandemic. In addition, once the pandemic subsides, a significant portion of patients will likely remain triaged to receive phone calls rather than in-person visits. Therefore, exploring the use and acceptance by patients and clinicians, including those who do not routinely conduct cognitive assessments, is key to further dissemination of remote preoperative cognitive screening.

## Materials and methods

This study was a prospective feasibility study of a telephone based cognitive evaluation in patients aged 70 years and above evaluated in the preoperative clinic who were scheduled to undergo a major elective surgery during December 2020 to March 2021 in a single tertiary care academic center. The study was approved by the appropriate Institutional Review Board (IRB# 2020P003086). Written informed consent was obtained from all subjects or their legal surrogates. In the preoperative clinic, all patients are routinely asked if they agree to be approached by a research team member, and only those who approved were called 1–3 days after their initial visit to obtain consent. The inclusion criteria included patients aged ≥ 70 year who were scheduled for any elective surgery, requiring postoperative admission to the hospital. Exclusion criteria included hearing impairment, day surgery, inability to speak English, and a prior diagnosis of dementia. The research team member also confirmed the participant had a primary care provider who can be contacted if any concerns regarding cognitive or functional impairment arise during the study period.

All members of the research team completed the online training course and certification on www.mocatest.org and therefore were officially certified to administer the MoCA test. Due to the required training and certification, we limited the number of co-investigators in this study to four (two certified geriatricians and two certified anesthesiologists). In addition, for quality assurance and standardization, prior to launching the study, the geriatricians on the team demonstrated how to conduct the telephone-based assessment and addressed any questions from the team. During the weekly meetings, the team discussed concerns and challenges of administering the preoperative cognitive evaluation over the phone and addressed specific questions such as reviewing the medication list and interpretation of specific parts of the test (mostly the word fluency test) for standardization. At the end of the study, we conducted a survey among the participating co-investigators using the Likert scale to evaluate their acceptance and perspectives on conducting this assessment over the phone. The surrogate for acceptance by patients was the completion of the full assessment, including the second assessment, conducted approximately 30 days after surgery. Our study aims were to determine in a pilot the feasibility of clinicians in the perioperative space to train to administer the test, how long does it take to conduct the test, and whether the test is acceptable for patients to complete perioperatively. As far as we are aware, there are no current publications outside of our group that this instrument has not been used in surgical populations.

### Study protocol and data collection

Eligible patients were called over the phone prior to their scheduled surgery, and the informed consent document was reviewed and virtually signed by a team member. A copy of the consent form was offered and sent by request. The study included the following steps:

1. Baseline demographics were collected including age, type of surgery, gender, ethnicity, and highest level of education.
2. All participants were asked a question regarding any concerns of memory impairment in the past 2 years.
3. Full list of medications including over-the-counter medi8cations and herbal supplements was collected. This list was later inserted into an anti-cholinergic calculator (ACS-acbcalc.com) to assess the total anticholinergic burden. In addition, the number of benzodiazepines and psychotropic medications was assessed and documented, including antipsychotic medications, selective serotonin reuptake inhibitors, serotonin-norepinephrine reuptake inhibitors, trazodone, mirtazapine, and tricyclic anti-depressants.
4. Functional status was evaluated using the Katz Index of Independence in Activities for Daily Living (ADL) and the Lawton-Brody Instrumental Activities of Daily Living Scale (IADL).
5. We screened for anxiety by using the Generalized Anxiety Disorder 2-item tool (GAD-2).
6. A brief test of attention was conducted and recorded, using the component of attention testing from the 3-minute diagnostic Confusion Assessment Method (3D-CAM) test (3D-CAM: derivation and validation of a 3-minute diagnostic interview for CAM-defined delirium: a cross-sectional diagnostic test study 2021), by asking the patient to recite the days of the week backwards.
7. The T-MoCA test was administered and timed. The T-MoCA is an abbreviated version of MoCA that does not require the use of a pencil and paper or visual stimulus and can be found on the official MoCA test website. It examines 5 cognitive domains including attention, verbal learning and memory, executive functions/language, and orientation (Wong et al. 2015). The total possible score of the version we administered was 15 points, with a score of 11 or more considered normal. The patient received a maximum of 4 points for verbal fluency, 6 points for orientation, and 5 points for delayed recall.

The entire telephone assessment was timed and a follow-up assessment, 30 days after the surgery was scheduled. In the 30-day follow-up assessment, the same questions and tools were applied, with an added opening question about any noted changes in memory or attention since surgery.

Study data was collected and managed using the Research Electronic Data Capture (REDCap) tools hosted at Mass General Brigham (Harris et al. 2019). This manuscript adheres to the applicable CONSORT guidelines.

### Statistical analysis

Continuous variables were analyzed using medians and interquartile range (IQR) or means and standard deviation (SD) accordingly.

## Results

We recruited 40 patients and completed perioperative cognitive assessments during the study period (December 2020 to March 2021). Demographics are detailed in Table 1. The cohort had a median age of 76 years (IQR 73-79), with 50% of patients being female and 97.5% white. Education level was high, as 35% of patients had an advanced degree and 47% had college education. The surgeries performed in this group were primarily orthopedic, cardiac, and thoracic/abdominal (27.5%, 22.5%, 17.5%, respectively). Before surgery, the median number of medications prescribed was 8 (IQR 7-11), with 25/40 (62.5%) taking medications with anticholinergic effects and a median anticholinergic score of 1 (IQR 0-2). Additionally, 6/40 (15%) were taking benzodiazepines, and 10/40 (25%) were taking psychotropic medications. The median Katz score was 6 (IQR 6-6), and Lawton-Brody score 8 (IQR 6-8). The mean time for completing the phone assessment was 10 min (IQR 8.25-12) and 4 min (IQR 3-5) for T-MoCA test. The median T-MoCA score was 13 (IQR 12-14) (Table 2).

**Table 1:** Baseline demographics

Variable	
**Gender—female*n*(%)**	20 (50%)
**Age, median (IQR)**	76 (73–79)
**Race—white*n*(%)**	39 (97.5%)
**Ethnic group—non-Hispanic*n*(%)**	36 (90%)
**Type of surgery**	
Orthopedic	11 (27.5%)
Cardiac	9 (22.5%)
Thoracic/abdominal	7 (17.5%)
**Education level**	
High school *n* (%)	7 (17.5%)
College *n* (%)	19 (47.5%)
Advanced degree *n* (%)	14 (35%)
**Medications—prior to surgery**	
Total number median (IQR)	8 (7–11)
Psychotropic medications *n* (%)	10 (25%)
Benzodiazepines *n* (%)	6 (15%)
Anti-cholinergic medication *n* (%)	25 (62.5%)
Anti-cholinergic score median (IQR)	1 (0–2)

**Table 2:** Summary of pre- and post-operative assessments

Variable	Pre-operative assessment (median, IQR)	Post-operative assessment (median, IQR)
Time for T-MoCA, minutes	4 (3–5)	3 (3–4)
T-MoCA score	13 (12–14)	13 (12–14)
Time for the full assessment, minutes	10 (8.25–12)	8 (7–12.25)
Katz score for ADL^a^	6 (6–6)	6 (6–6)
Lawton-Brody score for IADL^b^	8 (6–8)	8 (5–8)
GAD^c^ 2-item	1 (0–1)	0 (0–0)

^a^*ADL* Activities of Daily Living

^b^*IADL* Instrumental Activities of daily living

^c^*GAD* Generalized Anxiety Disorder, *T-MoCA* MoCA 5-minute test/Telephone

The postoperative cognitive assessment completion rate was 37/40 (92.5%) in the prespecified time frame, with a median of 36 days postoperatively (IQR 31-38). Of the three patients who did not complete the postoperative assessment, one died postoperatively, one did not undergo surgery, and one had his surgery postponed beyond the study period. During the postoperative assessment, 6/37 (16.2%) of patients reported that they had experienced a change in memory or attention since surgery. In this postoperative assessment, the median number of medications was 11 (IQR 7-13). Twenty-four of 37 (65%) of patients were taking anticholinergic medications with a median anticholinergic score of 1 (IQR 0-3), and 5/37 (16.2%) were taking benzodiazepines. The median Katz score was 6 (IQR 6-6) and Lawton-Brody score 8 (IQR 5-8). The mean time for completing the phone assessment was 8 min (IQR 7-12.25) and 3 min (IQR 3-4) for the T-MoCA test. The T-MoCA score was 13 (IQR 12-14), similar to the pre-operative assessment (Table 2).

All investigators reported in the weekly meetings that it was feasible for them to conduct the entire evaluation and T-MoCA test over the phone. A post-study survey demonstrated that most found the training easy to perform and were satisfied with the training provided by the official MoCA site. Investigators agreed that other clinicians, including those who do not routinely perform cognitive assessments, will be able to do so after completing the official training. In addition, most (75%) did not experience any patient declined doing the test itself, and all found the time to perform the test acceptable (see questionnaire responses, Table 3).

**Table 3:** Survey for clinicians conducting the study using the Likert scale (*n* = 4)

1. Prior to this study, have you conducted cognitive assessments on patients prior to surgery?	
50% yes, 50% no	
2. Overall, how satisfied or dissatisfied were you with the official training on the MoCA site (https://www.mocatest.org/)?	
50% very satisfied, 50% somewhat satisfied	
3. Did you find the training and following exam easy to perform?	
100% easy to perform	
4. Other clinicians, including those who do not routinely perform cognitive assessments, will be able to perform this test after completing the official MoCA training	
50% strongly agree, 50% agree	
5. Did you find patients were receptive to doing the tests?	
100% yes	
6. Did any patients decline doing the test?	
75% no, 25% yes due to anxiety regarding upcoming surgery, had other commitments, busy	
7. Did you find the time to perform the test acceptable?	
100% yes	
8. Please leave comments with any pros and cons of performing the test in this population	
“Easy to do after training and patients very receptive also patients found questions very understandable”	
“Pros- ease of use and short time it takes Cons- with high level of education it seems less sensitive to subtle changes, limited testing of executive functions”,	
“The population was highly educated, and this test may have been less sensitive to subtle cognitive changes. At the same time, it was a self-selected population who chose to participate in the study, which created an inherent bias. The pros of this test are that it's very short, easy to learn and use and may easily fit in the busy clinician's workflow. It is also very well suited for the phone assessment”,	
“The test was easy to administer, and patients did not find it time consuming either and were usually surprised the test was over as it went by quickly”	

## Discussion

We designed the study to explore the feasibility of using the T-MoCA test over the telephone to screen for cognitive impairment in the perioperative setting. This design involved determining whether clinicians from different specialties could quickly train to administer the test, if the time to conduct the test is reasonable, and if the test is acceptable for patients to complete before and after surgery. We demonstrate that T-MoCA test is easy to perform for clinicians and acceptable for patients in the perioperative period by showing high completion rates of both assessments and the ease of use by clinicians new to cognitive assessments. We have found that patients in our study (all age ≥ 70 years) can complete the test in less than 5 min while the overall encounter, including questions on medications, function, and anxiety screen, was completed in a median time of 8–10 min. In addition, we showed a high completion rate of the postoperative assessment (92.5%).

Previous studies have shown the importance of preoperative cognitive screening to evaluate cognitive impairment, which allows for mitigating postoperative complications such as delirium and postoperative cognitive decline (POCD) (Culley et al. 2017; Susano et al. 2020). These studies and others have led to a vast acceptance of the importance of cognitive screening in the preoperative period (Arias et al. 2020; Mahanna-Gabrielli et al. 2019; Cooper et al. 2020), though implementation remains challenging and limited (Rubin and Peden 2020). In addition, The Commonwealth of Massachusetts enacted a new law mandating all hospitals to implement an operational plan for early recognition and management of patients with dementia or delirium in acute care settings (Massachusetts-Health-Hospital-Association-Guidance-for-Developing-an-Operational-Plan-to-Address-Diagnosis.pdf 2021).

Our findings agree with the previously reported feasibility and reliability of administering a brief cognitive test over the phone. On the other hand, there are many other tools available for telephone-based cognitive assessments (Carlew et al. 2020), which are helpful not only in times of the pandemic for the safety of our patients and staff but for patients in remote areas as well, especially when coming to the hospital becomes more challenging. Available screening tests include *Telephone Interview for Cognitive Status (TICS)*, *animal fluency test*, *8 Item Screener (8-IS)*, *6- Item Cognitive test (6-CIT)*, and others (Lines et al. 2003; Lipton et al. 2003). TICS was one of the earliest cognitive screening tests for dementia developed for administration via telephone. The TICS demonstrated a 94% sensitivity and 100% specificity for cognitive impairment and was also highly correlated with the Mini-Mental State Examination (MMSE) (Desmond et al. 1994). TICS does well in distinguishing dementia from normal cognition but is less sensitive at detecting mild cognitive impairment (MCI). One advantage of the T-MoCA test is that it correlates to the full MoCA test, which is more sensitive to detection of MCI than the MMSE (Nasreddine et al. 2005) and was specifically shown to be more sensitive for MCI in stroke patients as compared to TICS (Cohen and Alexander 2017). Furthermore, it was also recently shown to be a sensitive screening tool for MCI in diverse community-dwelling populations, with a high correlation to a complete, in-person neuropsychological evaluation (Katz et al. 2021). Another advantage we noted is that while this test assesses multiple cognitive domains, it is quickly conducted over the telephone and does not require more advanced technology such as video or other applications. Phone-based cognitive assessment is critical in the older population since many do not access to more advanced technologies. A recent study showed that up to 40% of American older adults are not ready for telemedicine video-based visits (Lam et al. 2020). On the other hand, many have access to telephones, making these perioperative assessments acceptable and readily accessible, even in times of limited in-person visits.

The T-MoCA test was designed as a screening tool for cognitive impairment. It should be used to trigger further in-depth cognitive evaluation, not serve solely as a diagnostic tool for either MCI, dementia, or POCD. In medical centers that have accessible geriatric services, using this test for cognitive screening and further seeking geriatrics expertise can assure better utilization of resources effectively for patients and clinicians. While recognizing the limited access to geriatrics clinicians in many hospitals and preoperative clinics, using this cognitive screening tool can help implement pathways for high-risk older adults with suspected cognitive impairment in the postoperative period (Bryant et al. 2019). Our future research will focus on the association of specific preoperative cognitive assessment to postoperative outcomes and implementation pathways to mitigate these risks in older adults who are candidates for major surgery.

Our study adds new information to the limited work on using this screening tool preoperatively. The single study involving the use of this tool preoperatively did not use an investigator who actually had obtained certification in using the tool, as noted in their methods section (Yu et al. 2021). Certification involves learning modules as well as an exam and would obviously be essential to ensure appropriate use and validate results, so it is difficult to make any conclusion about the results of this study. The other two studies involve use postoperatively as well as use in a geriatric virtual clinic (O’Gara et al. 2020; Joughin et al. 2021). Our institution as well as a number of others are in the process of implementing geriatric surgery multidisciplinary care; however, the triage to which patients actually see a geriatrician and the limited geriatric resources do not make this a viable option for routine use.

### Limitations

Our study has several limitations. First, this is the experience of a single, academic medical center in a population of older adults with high education level, functional baseline and socio-demographic characteristics that may limit the generalization of our results to other populations. On the other hand, since we found it acceptable to both patients and clinicians, we assume this should not be a barrier for broad implementation in different, perhaps less selected, settings.

Second, patients aged 85 years and older were less represented in this cohort due to challenges in recruiting older adults to clinical research studies. We believe that once this screening tool is implemented as part of routine preoperative assessment and conducted during the scheduled appointment, these barriers will no longer exist.

Third, we did not include in this study other clinicians such as nurses, physician assistants, and nurse practitioners. However, we believe that once the appropriate training is completed and routine quality assurance measures are performed, this should not limit wide implementation in the preoperative setting.

## Conclusion

Preoperative cognitive assessment of older adults using T-MoCA over the phone is easy to perform for clinicians and had a very high patient completion rate. This test may be feasible for virtual assessments. Further research is needed to better define validity and association with postoperative outcomes.

## Data Availability

Please contact the author for data requests.

## Abbreviations

3D-CAM: 3-minute Diagnostic Confusion Assessment Method
6-CIT: 6-Item Cognitive test
8-IS: 8 Item Screener
ADL: Activities of Daily Living
ACS: Anti-Cholinergic Score Calculator
ASA: American Society of Anesthesiologists
COVID-19: Coronavirus disease-19
GAD-2: Generalized Anxiety Disorder 2-item
IADL: Instrumental Activities of Daily Living
IQR: Interquartile range
MCI: Mild cognitive impairment
MMSE: Mini-Mental State Exam
MoCA: Montreal Cognitive Assessment
POCD: Postoperative cognitive decline
REDCap: Research Electronic Data Capture
T-MoCA: Telephone Montreal Cognitive Assessment
TICS: Telephone Interview for Cognitive Status
